# Glomerular Properdin Deposition Suggests a Favorable Therapeutic Response to Tonsillectomy and Steroid Pulse Therapy in IgA Nephropathy

**DOI:** 10.7759/cureus.117956

**Published:** 2026-10-15

**Authors:** Tomoaki Miyazaki, Yuki Kajio, Akiko Takeshima, Kei Matsumoto

**Affiliations:** 1 Department of Medicine, Division of Internal Medicine (Nephrology), Showa Medical University Koto Toyosu Hospital, Tokyo, JPN

**Keywords:** complement alternative pathway, iga nephropathy, properdin, steroid pulse therapy, tonsillectomy

## Abstract

Background

Glomerular properdin deposition in IgA nephropathy (IgAN) indicates alternative pathway activation and is associated with severe histological activity. However, its impact on the therapeutic response to tonsillectomy combined with steroid pulse therapy (TSP) remains insufficiently elucidated.

Methods

We retrospectively evaluated 28 patients with primary IgAN who completed the TSP protocol. Patients were classified into properdin-positive (P+, n = 17) and properdin-negative (P-, n = 11) groups. We compared baseline clinicopathological features and clinical outcomes at one year, including clinical complete remission (CR) rates.

Results

At baseline, there were no significant differences in clinical parameters, including urinary protein and estimated glomerular filtration rate, between the two groups. However, histologically, the P+ group tended to present with more severe active inflammatory lesions, such as higher histological grades and more frequent crescent formation. One year after TSP, both groups showed significant reductions in urinary protein. Notably, despite their severe histological presentation, the P+ group achieved a median urinary protein reduction of -85.4% and a clinical CR rate of 58.8%, which were statistically equivalent to those in the P- group (-68.6% and 63.6%, respectively).

Conclusions

Although glomerular properdin deposition reflects severe active histological lesions in IgAN, it also indicates complement-driven acute inflammation that is highly susceptible to intensive immunosuppression. TSP effectively suppresses this properdin-associated tissue injury, achieving favorable clinical outcomes regardless of histological severity. Properdin evaluation may assist in positioning TSP as a highly cost-effective first-line strategy.

## Introduction

IgA nephropathy (IgAN) is the most common primary glomerulonephritis worldwide and a major cause of end-stage kidney disease [1]. In Japan, tonsillectomy combined with steroid pulse therapy (TSP) is widely performed and has been shown to induce clinical remission and protect long-term kidney function [2]. However, predicting individual treatment responses remains a clinical challenge due to the diverse clinicopathological presentations of the disease [3].

The complement system, particularly the alternative pathway (AP), plays a pivotal role in the pathogenesis and progression of IgAN [4]. Properdin, the specific positive regulator of the AP, functions by stabilizing C3 and C5 convertases on the cell surface, thereby significantly amplifying the complement cascade and exacerbating localized glomerular inflammation [5]. Consequently, complement AP activation, evidenced by glomerular properdin deposition, is strongly associated with highly active histological lesions, such as cellular or fibrocellular crescents, and is widely recognized as a surrogate marker for severe, progressive disease [6].

Despite this association with severe tissue injury, the impact of AP activation and properdin deposition on the therapeutic response to intensive immunosuppressive treatments like TSP remains insufficiently elucidated. Relying solely on conventional light microscopy to evaluate localized active lesions (e.g., endocapillary hypercellularity and crescents) is inherently limited by sampling errors [7]. In contrast, immunofluorescence allows for the objective molecular evaluation of complement activation throughout the entire tissue section. We hypothesized that while properdin deposition indicates intense, complement-driven acute inflammation, these active lesions might paradoxically exhibit high susceptibility to steroid therapy, representing a reversible state rather than irreversible fibrotic damage.

Therefore, this retrospective cohort study aimed to investigate the clinical significance of glomerular properdin deposition in patients with IgAN treated with TSP. We sought to determine whether properdin could serve as a useful, objective marker for evaluating post-treatment outcome associations, specifically our primary endpoints of clinical complete remission and proteinuria reduction, and for identifying candidates who might benefit from early therapeutic intervention.

## Materials and methods

Study design and eligibility criteria

This was a single-center, retrospective cohort study evaluating the clinical significance of glomerular properdin deposition on the therapeutic response to TSP in patients with IgAN. The study included patients who underwent a renal biopsy and were diagnosed with primary IgAN at Showa Medical University Koto Toyosu Hospital, Tokyo, Japan, between April 2018 and March 2025. Patients who did not receive the Sendai TSP protocol [8-10] (e.g., managed with conservative therapy alone), those with secondary IgAN (e.g., lupus nephritis, IgA vasculitis, hepatic IgA deposition), and those with incomplete clinical data were excluded. The study was approved by the Institutional Review Board of Showa Medical University Koto Toyosu Hospital (approval number: 2026-0266, dated July 31, 2026) and was conducted in accordance with the principles of the Declaration of Helsinki.

Treatment protocol

All included patients received TSP based on the Sendai protocol [8-10]. The timing of the tonsillectomy varied depending on individual clinical circumstances; it was performed either before the initiation of steroid pulse therapy or between the courses of steroid administration. The steroid protocol consisted of three courses of intravenous methylprednisolone (0.5 g/day for three consecutive days per course) administered weekly. Following the pulse therapy, oral prednisolone was initiated at a dose of 30 mg every other day. The dose was subsequently tapered by 5 mg every two months, resulting in complete discontinuation approximately one year after initiation.

Histopathological analysis

Renal biopsy specimens were evaluated using light microscopy, immunofluorescence (IF), and electron microscopy. Histopathological findings were scored according to the Oxford classification (MEST-C (Mesangial hypercellularity, Endocapillary hypercellularity, Segmental glomerulosclerosis, Tubular atrophy/interstitial fibrosis - Crescents) score) [3,7]. In addition, the overall disease severity and active inflammatory status were evaluated using the histological grade (H-grade: I to IV) according to the Japanese histological severity classification and the activity classification (A: acute, A/C: acute and chronic mixed, C: chronic) [11]. Glomerular properdin deposition was evaluated by direct immunofluorescence staining on frozen renal biopsy sections. Briefly, the sections were fixed with an ether-ethanol mixture for 10 minutes, followed by 95% ethanol for 20 minutes. After washing twice with phosphate-buffered saline (PBS), the sections were incubated with a fluorescein isothiocyanate (FITC)-conjugated anti-human properdin antibody (GAHu (goat anti-human)/properdin/FITC Cat. No. 16245; Nordic-MUbio, Susteren, the Netherlands) at a 1:2 dilution in PBS for 60 minutes. Following two additional PBS washes, the sections were mounted. Properdin positivity (P+) was strictly defined as a staining intensity of 1+ or greater, predominantly localized in the mesangial area. To ensure objective and reproducible evaluation, all IF samples were independently reviewed by two experienced nephrologists. Based on this consensus, the patients were divided into a properdin-positive (P+) group and a properdin-negative (P-) group.

Clinical and laboratory data assessment

Clinical data, including age, sex, estimated glomerular filtration rate (eGFR), urinary protein excretion (UP, g/gCr), and urinary red blood cells (U-RBC), were collected at baseline (at the time of renal biopsy) and strictly at the completion of the TSP protocol. The percentage reduction in UP was calculated using the following formula: $\frac{\text{Post-treatment UP} - \text{Pre-treatment UP}} {\text{Pre-treatment UP}} \times 100$. Clinical complete remission was defined in accordance with the established guidelines of the Japanese Society of Nephrology [12], representing the simultaneous achievement of both resolution of hematuria (U-RBC < 5/HPF) and a reduction in proteinuria to less than 0.3 g/gCr.

Statistical analysis

Continuous variables were expressed as median with interquartile range (IQR) and compared between groups using the Mann-Whitney *U* test. Intra-group comparisons (before and after treatment) were performed using the Wilcoxon signed-rank test. Categorical variables were compared using Fisher's exact test. A *p*-value of < 0.05 was considered statistically significant. All statistical analyses were performed using JMP Student Edition version 19.1.2 (SAS Institute Inc., Cary, North Carolina, United States).

## Results

Baseline clinical characteristics

A total of 73 patients were diagnosed with primary IgAN during the study period. After excluding 45 patients who did not receive the TSP protocol or met other exclusion criteria, 28 patients were included in the final analysis (Figure 1). The baseline clinical characteristics were well balanced between the two groups. There were no statistically significant differences in median age (P+ 44 vs. P- 41 years, *p* = 0.869) or the proportion of male patients (47.1% vs. 36.4%, *p* = 0.705). Renal function was also comparable, with a median eGFR of 73 mL/minute/1.73m² in the P+ group and 62 mL/minute/1.73m² in the P- group (*p* = 0.180). Although not statistically significant, the median baseline UP excretion showed a numerically higher trend in the P+ group (0.81 g/gCr) compared to the P- group (0.56 g/gCr) (*p* = 0.480) (Table 1).

**Figure 1:**
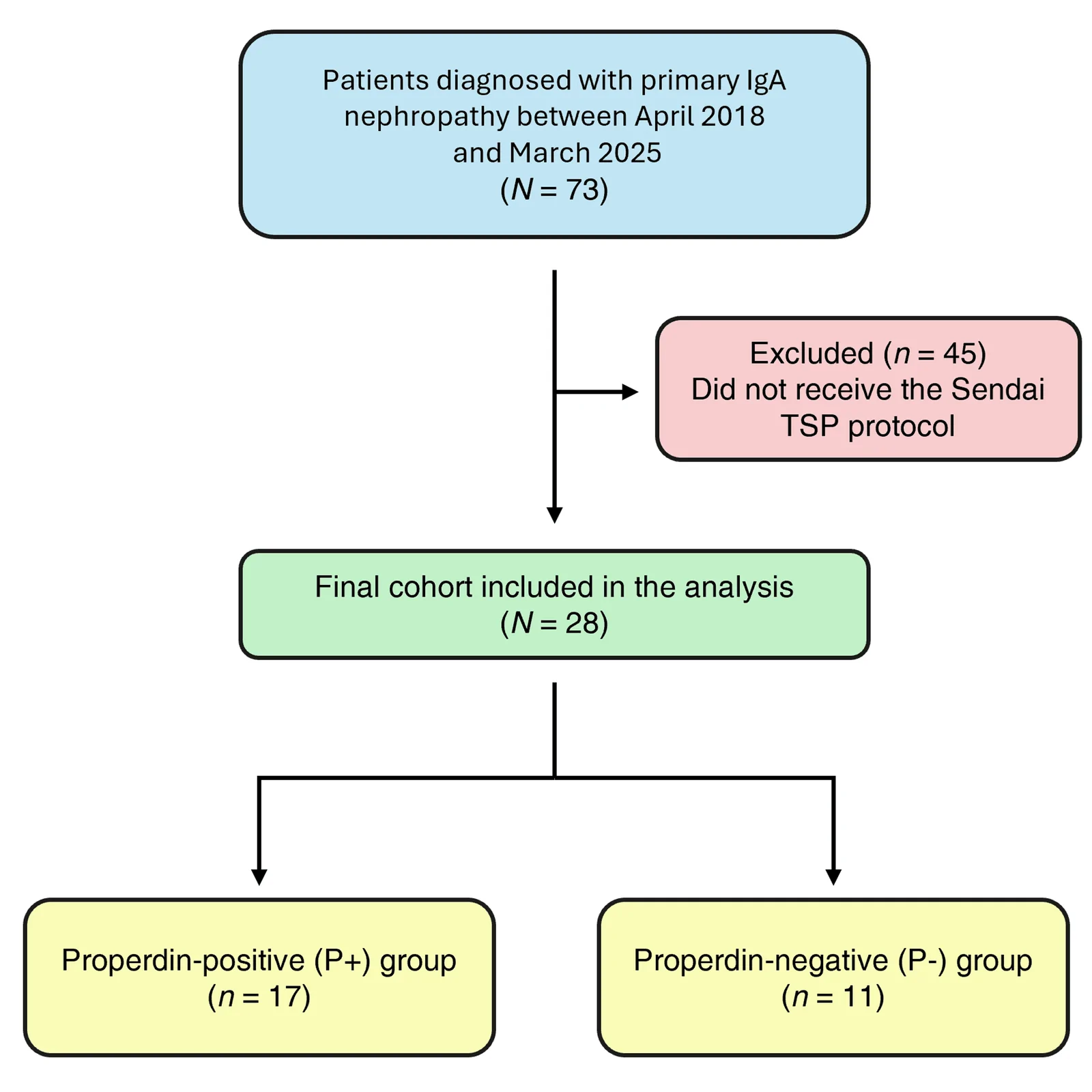
Study flow diagram detailing patient selection A total of 73 patients were diagnosed with primary IgA nephropathy between April 2018 and March 2025. After excluding 45 patients who did not receive the Sendai TSP protocol, 28 patients were included in the final analysis.The final cohort was divided into the properdin-positive (P+) group (*n* = 17) and the properdin-negative (P-) group (*n* = 11). IgAN: immunoglobulin A nephropathy; TSP: tonsillectomy combined with steroid pulse therapy; P+: properdin-positive; P-: properdin-negative.

**Table 1:** Baseline clinical characteristics Continuous variables are expressed as median (interquartile range). Categorical variables are expressed as count (percentage). *p*-values were calculated using the Mann-Whitney *U* test for continuous variables (test statistic: *Z*) and Fisher's exact test for categorical variables. Note: As Fisher's exact test calculates the *p*-value directly from the exact hypergeometric distribution, there is no corresponding test statistic (e.g., *t*, χ², or *F*) to report. eGFR: estimated glomerular filtration rate; HPF: high-power field; P+: properdin-positive; P-: properdin-negative; IQR: interquartile range

Clinical features	Total (N = 28)	P+ group (n = 17)	P- group (n = 11)	*p*-value	*Z*-value
Age (years), median (IQR)	43 (34.5-50)	44 (35-50)	41 (31-60)	0.869	0.16
Male, n (%)	12 (42.9%)	8 (47.1%)	4 (36.4%)	0.705	-
Creatinine (mg/dL), median (IQR)	0.86 (0.71-1.06)	0.85 (0.72-0.93)	0.9 (0.7-1.16)	0.452	0.75
eGFR (mL/min/1.73 m²), median (IQR)	65 (54.5-79.5)	73 (58.5-83)	62 (41-75)	0.180	-1.34
Urinary protein (g/gCr), median (IQR)	0.75 (0.39-1.16)	0.81 (0.45-1.44)	0.56 (0.30-1.08)	0.480	-0.71
Microscopic hematuria (≥ 5 /HPF), n (%)	26 (92.9%)	17 (100%)	9 (81.8%)	0.146	-

Baseline histopathological characteristics

Regarding the histopathological evaluation, the P+ group demonstrated a tendency toward more severe and active inflammatory lesions compared to the P- group; however, these numerical trends did not reach statistical significance, likely due to sample size constraints. Specifically, advanced tissue injury, defined as H-grade ≥ II, was observed in 35.3% of the P+ group versus 9.1% of the P- group (*p* = 0.191). Within the Oxford classification parameters, mesangial hypercellularity (M1) and endocapillary hypercellularity (E1) were present in 29.4% and 23.5% of the P+ group, respectively, showing a higher trend than the P- group (18.2% and 18.2%, respectively). Additionally, cellular/fibrocellular crescents (C1/C2) were observed in 58.8% of the P+ group compared to 45.5% of the P- group (*p* = 0.700). Conversely, the chronicity indices, including segmental glomerulosclerosis (S1) and tubular atrophy/interstitial fibrosis (T1/T2), were comparable between the cohorts (Table 2).

**Table 2:** Baseline histopathological characteristics *p*-values were calculated using Fisher's exact test. Note: As Fisher's exact test calculates the *p*-value directly from the exact hypergeometric distribution, there is no corresponding test statistic (e.g., *t*, χ², or *F*) to report. P+: properdin-positive; P-: properdin-negative; M: mesangial hypercellularity; E: endocapillary hypercellularity; S: segmental glomerulosclerosis; T: tubular atrophy/interstitial fibrosis; C: cellular/fibrocellular crescents; H-grade: histological grade; MEST-C: mesangial hypercellularity, endocapillary hypercellularity, segmental glomerulosclerosis, tubular atrophy/interstitial fibrosis - crescents

Histopathological features	Total (N = 28), n (%)	P+ group (n = 17) , n (%)	P- group (n = 11) , n (%)	p-value
Oxford MEST-C classification				
M1	7 (25.0%)	5 (29.4%)	2 (18.2%)	0.668
E1	6 (21.4%)	4 (23.5%)	2 (18.2%)	1.000
S1	7 (25.0%)	4 (23.5%)	3 (27.3%)	1.000
T1/T2	3 (10.7%)	2 (11.8%)	1 (9.1%)	1.000
C1/C2	15 (53.6%)	10 (58.8%)	5 (45.5%)	0.700
Japanese histological severity classification				
H-grade ≥ Ⅱ	7 (25%)	6 (35.3%)	1 (9.1%)	0.191
Activity (A or A/C)	15 (53.6%)	10 (58.8%)	5 (45.5%)	0.700

Therapeutic response to TSP

Despite presenting with more severe inflammatory histopathological features at baseline, the P+ group achieved a favorable therapeutic response following the completion of the TSP protocol, which was fully comparable to that of the P- group. At the time of evaluation, both groups demonstrated a substantial reduction in UP, with the P+ group reaching a median UP of 0.10 g/gCr (an 85.4% median reduction) and the P- group reaching 0.20 g/gCr (a 68.6% median reduction) (*p* = 0.778 for post-treatment UP, and *p* = 0.221 for percentage reduction). Moreover, the resolution of microscopic hematuria was achieved in 82.3% of the P+ group and 72.7% of the P- group (*p* = 0.653). Ultimately, the rate of CR (defined as the simultaneous resolution of hematuria and reduction of UP to < 0.3 g/gCr) was high and statistically equivalent between the two groups (58.8% in the P+ group vs. 63.6% in the P- group, *p* = 1.000) (Table 3). Furthermore, renal function remained stable in both groups, with no significant difference in median post-treatment eGFR between the P+ group (67.0 mL/minute/1.73m²) and the P- group (61.0 mL/minute/1.73m²) (*p* = 0.888), confirming the overall renal safety of the intensive immunosuppressive protocol.

**Table 3:** Clinical outcomes at the completion of the TSP protocol *p*-values were calculated using the Mann-Whitney *U* test for continuous variables (test statistic: *Z*) and Fisher's exact test for categorical variables. Note: As Fisher's exact test calculates the *p*-value directly from the exact hypergeometric distribution, there is no corresponding test statistic (e.g., *t*, χ², or *F*) to report. Clinical complete remission was defined as the simultaneous resolution of hematuria and reduction of urinary protein to < 0.3 g/gCr. eGFR: estimated glomerular filtration rate; UP: urinary protein; TSP: tonsillectomy combined with steroid pulse therapy; P+: properdin-positive; P-: properdin-negative.

Parameters	P+ group (n = 17)	P- group (n = 11)	*p*-value	*Z*-value
Post-treatment eGFR (mL/minute/1.73m²), median (IQR)	67 (53-80.5)	61 (52-80)	0.888	-0.14
Change in eGFR (mL/minute/1.73m²), median (IQR)	-3 (-8.5 to 1.5)	5 (-10 to 17)	0.144	1.46
Urinary protein at completion (g/gCr), median (IQR)	0.10 (0.05 to 0.49)	0.20 (0.05 to 0.35)	0.778	-0.28
Percentage reduction in UP (%), median (IQR)	-85.4 (-89.3 to -75.8)	-68.6 (-91.4 to -31.1)	0.221	1.22
Resolution of hematuria, n (%)	14 (82.3%)	8 (72.7%)	0.653	-
Clinical complete remission, n (%)	10 (58.8%)	7 (63.6%)	1.000	-

## Discussion

This retrospective cohort study investigated the clinical significance of glomerular properdin deposition in patients with IgAN who completed the Sendai TSP protocol. The major finding of this study is that although P+ patients tended to present with more severe and active histopathological features-such as cellular/fibrocellular crescents and a higher H-grade-their baseline clinical parameters, including proteinuria and eGFR, were comparable to those of the P- group. Furthermore, despite these highly active histological lesions, P+ patients demonstrated a favorable therapeutic response to TSP, achieving clinical outcomes that were fully equivalent to those of the P- group.

The favorable response to TSP in the P+ group may be explained by the upstream immunomodulatory effects of this therapy. While glomerular properdin deposition reflects downstream local complement amplification, TSP targets the mucosal immune system, which is considered the origin of the disease. Tonsillectomy is believed to reduce the production of galactose-deficient IgA1 (Gd-IgA1), and systemic corticosteroid administration suppresses subsequent immune complex formation and widespread inflammation [13]. By inhibiting the upstream trigger, the downstream complement cascade, including AP activation, may be halted before irreversible fibrotic damage occurs. Furthermore, the P+ group in our study exhibited higher rates of active inflammatory lesions, such as E1 lesions. Previous studies validating the Oxford classification have demonstrated that these acute active lesions, while indicative of severe ongoing injury, are responsive to immunosuppressive therapies [14,15]. This clinical characteristic aligns with our observation of rapid UP reduction following TSP in the P+ group.

Previous studies have established that alternative pathway activation, evidenced by properdin deposition, is strongly associated with crescent formation and a progressive clinical course in IgAN [6]. Historically, such highly active tissue manifestations have been regarded as indicators of poor long-term renal prognosis and potential treatment resistance [3,7]. However, our results highlight a critical discordance between histological severity and treatment responsiveness in the context of intensive immunosuppression. We hypothesize that glomerular properdin deposition reflects severe, complement-driven acute inflammation rather than irreversible chronic damage. High-dose systemic corticosteroid pulse therapy may effectively suppress this intense inflammation. This may explain why the P+ group, despite having histologically severe lesions, achieved a substantial median reduction in urinary protein of -85.4% without showing treatment refractoriness. Thus, properdin-associated active inflammatory lesions strongly reflect a state of high steroid susceptibility, identifying these patients as suitable candidates for early TSP intervention to prevent the progression to irreversible sclerosis.

Identifying patients who are candidates for TSP is clinically important. Although the Oxford classification (MEST-C score) is useful for predicting prognosis [3], the evaluation of localized active lesions, such as E and C lesions, by light microscopy can be affected by sampling errors during renal biopsy [7]. In contrast, immunofluorescence staining for properdin can robustly evaluate complement activation in the glomeruli, thereby complementing light microscopic findings. Therefore, properdin deposition is considered a useful and objective marker reflecting active lesions that may benefit from early TSP intervention. Recently, novel complement-targeted therapies, such as the alternative pathway inhibitor iptacopan, have demonstrated promising efficacy in IgAN [16]. While these emerging agents represent a significant therapeutic advancement, our findings highlight that TSP, a well-established and widely accessible conventional therapy, can suppress alternative pathway-driven inflammation and achieve clinical remission in P+ patients. Thus, evaluating glomerular properdin deposition is crucial not only for assessing histological activity but also for positioning TSP as a viable first-line strategy [17].

This study has several limitations. First, it is a single-center, retrospective study with a small sample size (n = 28). As Showa Medical University Koto Toyosu Hospital is not a high-volume center, the number of eligible patients was limited. This severely limits our statistical power, posing a risk for Type II errors when comparing baseline differences (e.g., eGFR, *p* = 0.180; H-grade ≥ II, *p* = 0.191). Second, the single-arm retrospective design lacks an untreated control group, meaning this study evaluates post-treatment outcome associations rather than definitive predictive treatment interactions. Third, there were inherent methodological variations, such as the unstandardized timing of tonsillectomy relative to steroid pulses, and a universally standardized diagnostic cutoff for properdin positivity in this clinical context has yet to be established. Finally, the follow-up evaluation occurred strictly at the completion of the TSP protocol to assess the acute therapeutic response. Further large-scale, multicenter prospective studies with longer observation periods are needed to evaluate the long-term preservation of renal function and establish the precise role of properdin in clinical practice.

## Conclusions

This study demonstrates that glomerular properdin deposition in IgAN is closely associated with severe and active histopathological lesions, including advanced H-grades and E1 lesions. However, our findings indicate that these P+ patients are not refractory to early immunomodulatory intervention. Following the completion of the Sendai TSP protocol, the P+ group achieved substantial reductions in UP and high rates of CR that were statistically equivalent to those observed in the P- group.

These results suggest that properdin deposition should not be viewed solely as a predictor of a poor long-term prognosis, but rather as an indicator of acute, severe inflammation that remains highly responsive to appropriately timed upstream treatments like TSP. In clinical practice, identifying P+ status may help stratify patients who would benefit most from prompt TSP initiation before irreversible fibrotic damage occurs. Further prospective, multicenter studies with extended observation periods are necessary to confirm these acute therapeutic responses and to establish the definitive role of properdin in evaluating treatment outcomes and guiding therapeutic selection.
